# Combinatorial Signal Integration by APETALA2/Ethylene Response Factor (ERF)-Transcription Factors and the Involvement of AP2-2 in Starvation Response

**DOI:** 10.3390/ijms13055933

**Published:** 2012-05-16

**Authors:** Marc Oliver Vogel, Deborah Gomez-Perez, Nina Probst, Karl-Josef Dietz

**Affiliations:** Biochemistry and Physiology of Plants, Bielefeld University, 33501 Bielefeld, Germany; E-Mails: marc_oliver.vogel@uni-bielefeld.de (M.O.V.); debsgp@gmail.com (D.G.-P.); nina.probst@gmx.net (N.P.)

**Keywords:** abscisic acid, apetala2/ethylene response factor, *Arabidopsis thaliana*, germination, photosynthesis, retrograde signaling, transcription factor

## Abstract

Transcription factors of the APETALA 2/Ethylene Response Factor (AP2/ERF)- family have been implicated in diverse processes during development, stress acclimation and retrograde signaling. Fifty-three leaf-expressed AP2/ERFs were screened for their transcriptional response to abscisic acid (ABA), 3-(3,4-dichlorophenyl)-1,1-dimethylurea (DCMU), methylviologen (MV), sucrose and high or low light, respectively, and revealed high reactivity to these effectors. Six of them (AP2-2, ARF14, CEJ1, ERF8, ERF11, RAP2.5) were selected for combinatorial response analysis to ABA, DCMU and high light. Additive, synergistic and antagonistic effects demonstrated that these transcription factors are components of multiple signaling pathways. AP2-2 (At1g79700) was subjected to an in depth study. *AP2-2* transcripts were high under conditions linked to limited carbohydrate availability and stress and down-regulated in extended light phase, high light or in the presence of sugar. *ap2-2* knock out plants had unchanged metabolite profiles and transcript levels of co-expressed genes in extended darkness. However, *ap2-2* revealed more efficient germination and faster early growth under high sugar, osmotic or salinity stress, but the difference was abolished in the absence of sugar or during subsequent growth. It is suggested that AP2-2 is involved in mediating starvation-related and hormonal signals.

## 1. Introduction

Plants encounter stress if abiotic growth parameters severely deviate from optimum and also during adverse biotic interactions. To avoid or minimize stress and to maintain high fitness, plants evolved mechanisms to acclimate to changing environmental conditions. The adjustment occurs on different regulatory and metabolic levels and usually involves cellular signaling pathways and transcription factors (TFs) which alter gene expression. The *Arabidopsis thaliana* genome encodes approximately 2000 TFs [1,2] categorized in distinct families. The Apetala2/ethylene response factor (AP2/ERF-TF) family [3,4] comprises nearly 7% of all TFs in *A. thaliana*. Database searches reveal 147 gene loci [5,6]. AP2/ERF-TFs are also found outside the plant kingdom [7]. In plants they are involved in signaling processes, e.g., ABI4 affects abscisic acid (ABA), redox and sugar signaling and acts downstream of genomes uncoupled 1 (GUN1) [8]. Another TF of this family, RRTF1, strongly responds to environmental stresses like high light [9]. DREB1A and DREB2A mediate transcriptional responses to cold, drought and salinity. They were identified in a yeast-one-hybrid screen with DRE sequences as bait (dehydration response element), which had been shown to mediate dehydration- and low temperature-induced responses. DREB1A was found to be cold-responsive and DREB2A drought-responsive, respectively [10]. Apparently both TFs function in two different signaling pathways despite their high similarity. Sakuma *et al.* [11] suggested an integrative model of signal transduction with DREB1A and 2A controlling three sets of genes with significance in abiotic stress acclimation [11]. Despite good knowledge on some AP2/ERF-TFs, the functions of most group members remain elusive.

Signal transduction during abiotic stress involves signaling pathways that are highly interlinked, assemble to complex networks and enable crosstalk [12,13]. Molecular interactions gradually or digitally elicit, enhance or suppress specific responses within a signaling network and, in transcriptional regulation, control TFs. Well-studied examples are the ABA-depended and the ABA-independent pathways in drought signaling [14,15] and in the related cold-response pathway [16,17] which besides AP2/ERF-TFs like RAP2.1 also involve other stress-related proteins like COR47 or ZAT12 [18].

Chloroplasts and in particular photosynthesis-linked cues take part in the cellular stress signaling network. In principle, organellar effects on nuclear gene expression mediated through retrograde signaling can be assigned to either developmental or operational control [19]. Thus, retrograde signaling is defined as communication between plastids and nucleus to adjust metabolic performance of the organelles to the cellular needs. The adjustment is mainly achieved by balancing synthesis of plastome-encoded and nuclear genome-encoded plastid proteins [20,21]. It is apparent that the operational type of retrograde control is intimately linked to stress acclimation, e.g., when conditions of excess excitation energy coincide with limited acceptor availability under drought or in coldness. Singlet oxygen, hydrogen peroxide, plastoquinone redox state, NADPH, ABA, lipid peroxidation products and sugars represent signals discussed in context of retrograde signaling [22–24]. Signaling processes triggered by these signals allow for acclimation of the photosynthetic apparatus to a changing and possibly stressful environment. This involves either the travelling of signaling molecules from plastids to nucleus or other mechanisms of information transfer. Combinatorial patterns of signals likely optimize the nuclear response [24]. Independent on the nature of the signals, they all alter gene expression of nuclear genome-encoded chloroplast proteins but also are involved in other stress acclimation responses.

AP2/ERF-TFs are involved in many signaling processes and stress responses [6] and, thus, represent promising candidates to also link retrograde signaling and stress acclimation as previously demonstrated for ABI4 [8]. The aim of this work was to address the involvement of AP2/ERF-TFs in the integration of signaling pathways that possibly are implicated in the retrograde control of nuclear gene expression. To this end, single and combinations of effectors were first tested on regulation of AP2/ERF-TF transcript level. The particularly responsive AP2/ERF-TF AP2-2 was selected for further investigations. *AP2-2* transcript regulation could be shown to be related to carbohydrate status and the absence of AP2-2 affected germination under stress.

## 2. Results

### 2.1. Effector Treatments with Relevance for Retrograde Signaling

**Figure 1:**
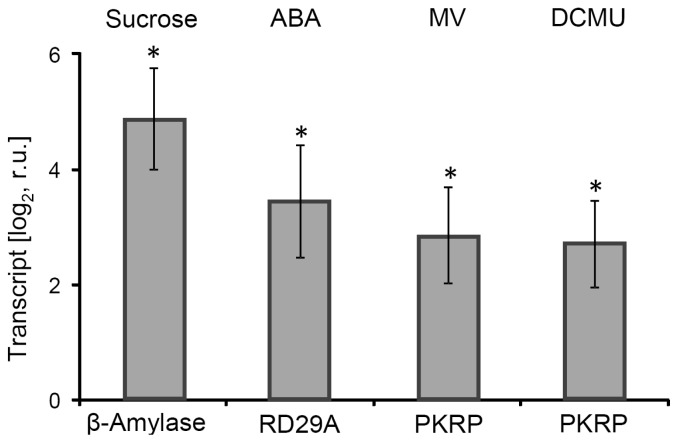
Transcript levels of marker genes in response to effector treatments. Leaf discs floated in 0.1 mM CaCl_2_ solution supplemented with 30 μM abscisic acid (ABA), 10 μM 3-(3,4-dichlorophenyl)-1,1-dimethylurea (DCMU), 1 μM MV and 30 mM sucrose, respectively, or without additions as control for 4 h. Marker transcripts responsive to sugar (β-amylase), ABA (RD29) or ROS (H_2_O_2_) (PKRP) were quantified and expressed as log fold change relative to control. Strong and significant >2.5 fold up regulation was seen for each selected marker. Data are means ± SE of three biological experiments (sqRT-PCR), *p*-value < 0.05, Student *t* test, normalized to the control with *actin2*.

The starting point of our work was to investigate the possible involvement of AP2/ERF-TFs in nuclear responses to retrograde signaling. To this end, leaf discs were treated with effectors which mimic retrograde signals or stimulate their release. Leaf discs were chosen in order to guarantee homogenous and rapid effector uptake. Discs floated upside down on 0.1 mM CaCl_2_ to allow for stomatal gas exchange. The first experiments aimed at confirming the effectiveness of sucrose, ABA, MV and DCMU in the leaf disc system. Therefore, marker transcript levels were quantified after 4 h of treatment. Selected marker genes were β-amylase (At4g15210) for sucrose accumulation [25], RD29a (At5g52310) for ABA treatment [26] and PKRP (pyruvate kinase related protein: At3g49160) for ROS-formation [27]. All marker genes showed the expected up-regulation with significant log_2_FC > 2.5 relative to non-treated control leaf discs (Figure 1). Chl a-fluorescence parameters were assessed to determine the quantum yield of photosystem II (ΦPSII). Low light (L) had no significant influence on ΦPSII whereas high light (H) led to a significant decrease by 43% after 1 h and 57% after 3 h of treatment (Figure S.1A). Sucrose as well as ABA had no and only a small effect on ΦPSII, respectively (Figure S.1B). After DCMU treatment ΦPSII decreased to 32% within 1 h and remained on this low level during the whole experiment. MV inhibited ΦPSII by 29% after 4 h (Figure S.1C). These data on ΦPSII combined with the transcriptional response of selected marker genes confirmed the usefulness of the treatments to manipulate retrograde signaling-related conditions.

**Figure 2:**
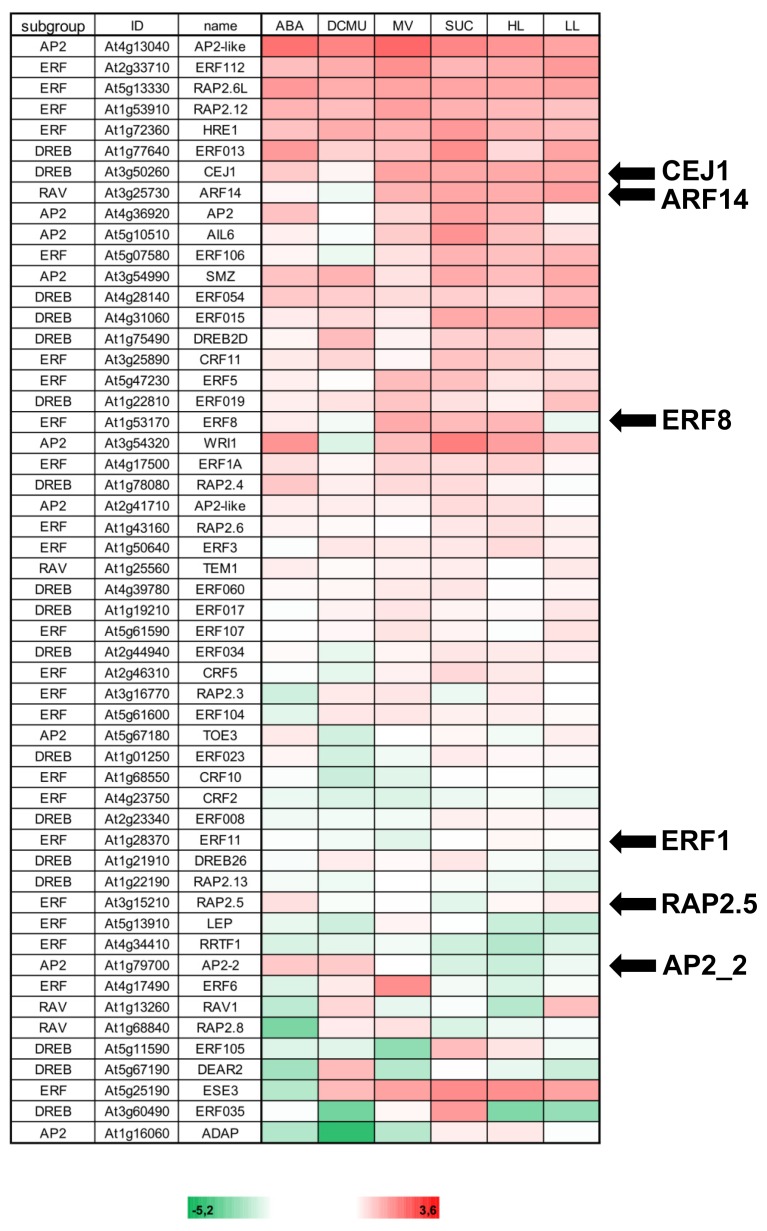
Transcript profiling of selected APETALA 2/Ethylene Response Factor Transcription Factors (AP2/ERF-TFs). Transcript levels of 53 AP2-ERF-TFs were analyzed by sqRT-PCR for their response to different effector treatments (30 μM ABA, 10 μM DCMU, 1 μM MV, 30 mM sucrose, high light (HL) and low light (LL) for 4 h using leaf discs floating on 0.1 mM CaCl_2_). The results served as a first screen to select interesting candidates for investigation of combinatorial regulation. Data were obtained from two independent biological experiments and are presented as log_2_-fold change (green color means down-regulation, red color up-regulation, respectively) following normalization to *actin2*. Data of selected TFs represent data from *n* = 6 experiments for DCMU and ABA.

This experimental design was employed to evaluate the influence of the effectors on transcript accumulation of selected AP2/ERF-TFs. To be involved in retrograde signaling from chloroplast to nucleus, the respective TF must be expressed in leaves. The work by Feng *et al.* [28] provides a mostly comprehensive set of transcript profiles for the whole AP2/ERF-TF family. From this work and by additional database searches 53 AP2/ERF-TFs were selected that are expressed in leaves and belong to all different subgroups of the AP2/ERF-TF family (Figure 2). Transcript level regulation relative to control was quantified by sqRT-PCR. This first experiment aimed at identifying candidates with interesting response behavior which could be selected for subsequent combinatorial analysis. Overall, the transcriptional changes of the 53 AP2/ERF-TFs were highly diverse and ranged between log_2_ FC − 5.2 to log_2_ FC + 3.6. Diversity of responses was seen within each subgroup of AP2/ERF-TFs and with respect to the 6 different treatments. Cluster analysis revealed a rather homogeneous up- and down regulation of some AP2/ERF-TFs to many treatments. Only few TFs showed a rather specific and strong response to a particular stimulus, e.g., *ERF At1g15210* and *RAV At1g68840* to ABA, *AP2 At1g16060* to DCMU, or *ERF At4g17490* to MV and *DREB At3g60490* to sucrose. But in general, the TF transcript levels responded to several stimuli. This analysis suggested multiple roles of AP2/ERF-TFs in retrograde and stress-related signaling.

### 2.2. Effector Concentration-Dependent Expression of AP2/ERF-TFs

**Figure 3:**
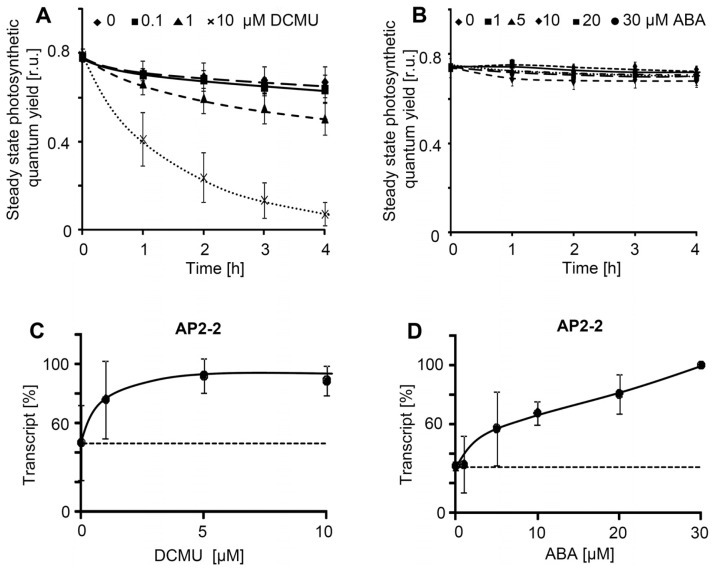
DCMU and ABA concentration dependencies of ΦPSII and expression levels of AP2-2. Chlorophyll a fluorescence analysis of leaf discs was used to quantify the quantum yield of photosystem II. In identically treated discs, AP2-2-transcript levels were quantified by sqRT-PCR. Data were taken from three experiments with six leaf discs in each experiment, ±SD.

Concentration-dependent responses were determined to characterize the dose-effect-relationship for ABA and DCMU. ΦPSII was measured to confirm the usefulness of the selected concentrations. ABA (1 to 30 μM) had no effect on ΦPSII (Figure 3), like DCMU at very low concentrations (<0.1 μM). At higher concentrations between 1 and 10 μM, DCMU decreased ΦPSII in a time-dependent manner. In parallel *AP2-2* transcript levels were almost steadily up-regulated with increasing ABA, while it reached saturation after application of DCMU at low concentrations (Figure 3).

### 2.3. Combinatorial Effects on AP2/ERF-TF Expression

Plants in the environment are often exposed to combinations of different stressors rather than to single ones. In such a multifactorial environment signal integration and cross talk evolved as fundamental property of plant signaling networks [12,29]. As shown in Figure 2, transcripts of leaf-expressed AP2/ERF-TFs respond to various stimuli related to retrograde and stress signaling. Therefore, we were interested in combinatorial responses. Six AP2/ERF-TFs were selected based on their transcriptional response in the experiments to Figure 2, particularly for their differential sensitivity to ABA and DCMU. *CEJ1* and *ARF14* clustered among the highly responsive AP2/ERF-TFs with opposite regulation to ABA. *ERF8* transcript differentially changed between L- and H-light. *ERF11*-mRNA decreased with DCMU, while *RAP2.5* transcript level was up with ABA and *AP2-2* both with ABA and DCMU.

**Figure 4:**
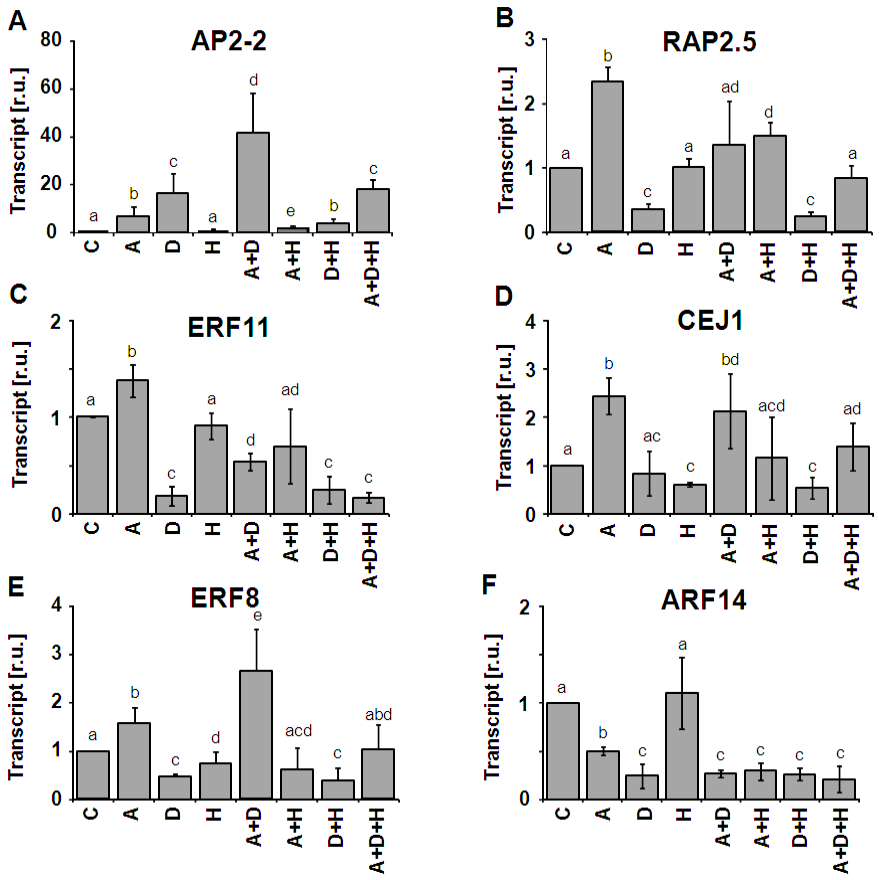
Transcript regulation of six AP2/ERF-TFs in response to combinatorial effector application. Leaf discs were treated with 30 μM ABA (A), 10 μM DCMU (D) and high light (H), respectively, and each combination. (**A**) *AP2-2*; (**B**) *RAP2.5*; (**C**) *ERF11*; (**D**) *CEJ1*; (**E**) *ERF8*, and (**F**) *ARF14*. The qRT-PCR data were obtained from *n* = 3 experiments with technical replicates and are presented as means ± SD. Identical letters indicate groups of same significance level (*p* < 0.05, Student *t* test).

Transcript levels of these six selected AP2/ERF-TFs were quantified by qPCR for their response to ABA, DCMU and H-light and each combination of these three treatments after 4 h, respectively (Figure 4). The aim was to identify synergistic and antagonistic effects and thereby multifactorial mechanisms in the signaling pathways. The expression level of *AP2-2* TF was strongly up-regulated by ABA or DCMU. The combination of both effectors acted strongly synergistic with an about 2-fold higher induction if compared to a purely additive response (Figure 4A). Interestingly H-light had no effect as single treatment, but strongly suppressed DCMU and ABA-induced up-regulation and also partly the combinatorial synergistic effect of DCMU plus ABA.

Each selected transcription factor mRNA responded in a distinct pattern (Figure 4). *RAP2.5*-transcript accumulation was increased in ABA-treated samples, but suppressed by DCMU. H-light had no effect. DCMU-dependent suppression compensated for ABA-induced stimulation. This contrasts regulation of *CEJ1* which was stimulated by ABA, unaffected by DCMU and slightly suppressed by H-light. Each combination realized the respective *CEJ1*-transcript level as expected by combining the single effects. The inhibitory effect of DCMU dominated regulation of *ERF11* and *ARF14*. The difference between both was that *ERF11* accumulation was slightly stimulated by ABA while *ERF11* transcript decreased in ABA-treated leaves. *ERF8* transcript behaved like *CEJ1* except for the more prominent stimulation of *CEJ1* by ABA.

### 2.4. AP2-2 Transcript Regulation Relates to Starvation and Stress Versus Satiety

**Figure 5:**
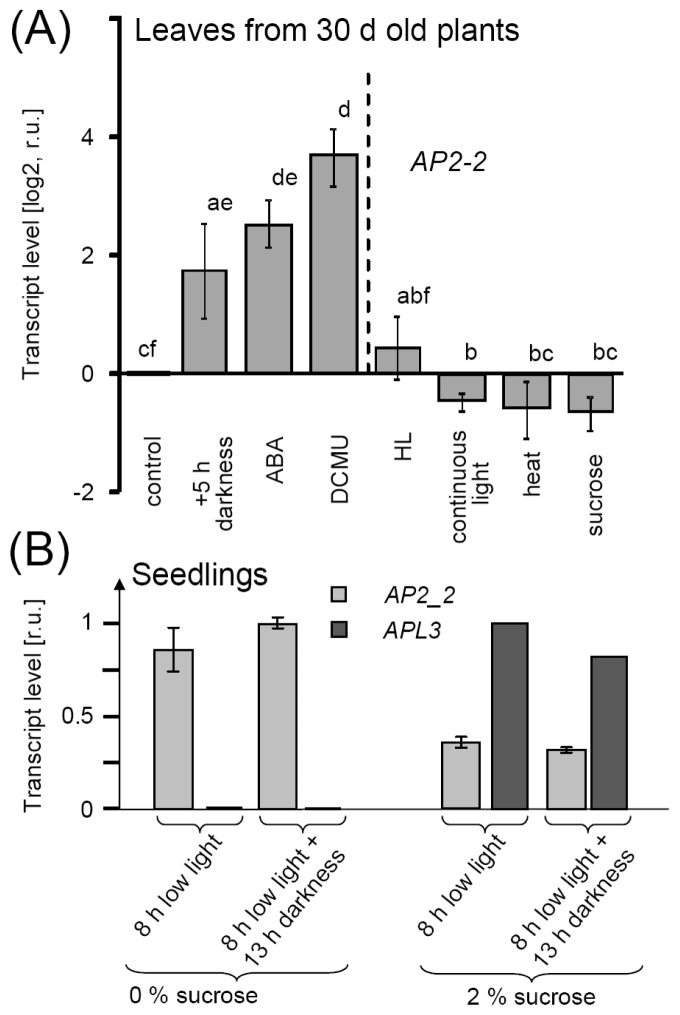
(**A**) Response of *AP2-2* to single effector application. *AP2-2* transcript level increased upon treatments which induce or indicate metabolic demand like extended darkness (5 h over normal), 30 μM ABA or 10 μM DCMU. In contrast, no reaction or even down regulation occurred under conditions encompassing metabolic and energy abundance, *i.e.*, high light, continuous light, heat treatment or sucrose (30 mM). The qRT-PCR data are means of *n* = 3 ± SE. Identical letters indicate same significance groups (*p* < 0.1, Student *t* test); (**B**) Up-regulation of *AP2-2*-transcript in low light or darkness with and without sugar supplementation. *AP2-2-*transcript was quantified in 13 days old seedlings by qRT-PCR at low light (8 μmol quanta·m^−2^·s^−1^) or at the end of the subsequent dark period. Seedlings were grown at 80 μmol quanta·m^−2^·s^−1^ in 0.25% sucrose/MS medium for 11 days and transferred to either MS medium with 0% or 2% sucrose. Samples were taken 2 days after transfer to the new conditions. Data are means of *n* = 4 from two independent experiments, ±SE.

*AP2-2* was selected for more detailed analysis because of the very strong response of its transcript to ABA and DCMU. ABA induces stomatal closure [30] and DCMU inhibits reduction of plastoquinone within the photosynthetic electron transport chain, thus, both inhibit photosynthesis and assimilate production by different mechanisms. Therefore, the stimulatory effect of both effectors on *AP2-2*-transcript accumulation was tentatively hypothesized to be linked to assimilate deficiency. In line with this hypothesis, extended darkness also increased *AP2-2*-mRNA levels, while sucrose feeding and continuous illumination caused *AP2-2*-mRNA depletion (Figure 5A). Heat stress decreased *AP2-2* mRNA accumulation. Transcript levels were also quantified in seedlings on sugar-free or sugar-containing MS medium (Figure 5B). In each case, sugar supplementation suppressed AP2-2-accumulation, while the sugar marker gene *APL3*, encoding the large subunit of chloroplast ADP glucose pyrophosphorylase for starch synthesis, responded as expected in the opposite way with low mRNA levels in sugar-free grown seedlings and high levels in plants grown on sugar-containing medium independent on darkness or L-light illumination. It is concluded that *AP2-2* transcript amounts tentatively and inversely correlate with assimilate availability.

### 2.5. AP2-2 Knock Out Plant is Disturbed in Seeding Establishment

**Figure 6:**
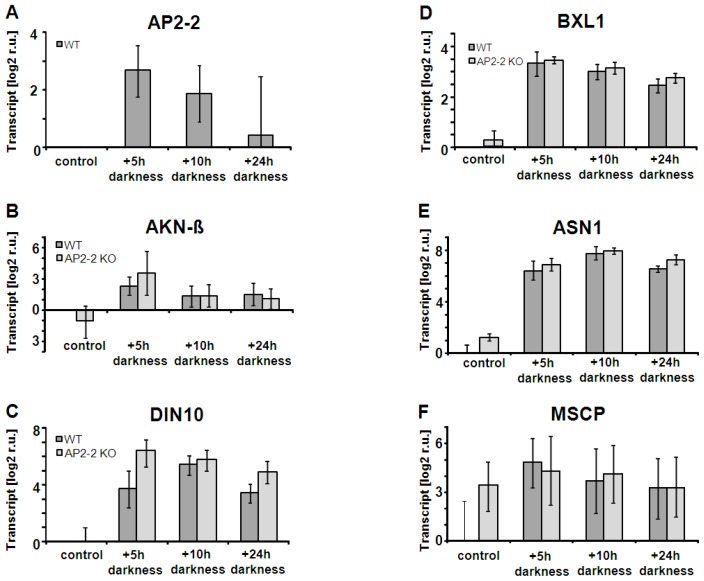
Regulation of transcripts identified as co-regulated with *AP2-2* in wild type and *ap2-2* knock out plants. Bioinformatic analysis of publicly available transcriptome data generated a gene list of co-regulated transcripts (Table S.2). Among the top 13 genes in the list were β-xylosidase 1 (BXL1), a protein kinase (AKINβ), dark induced raffinose synthase family protein (DIN10), a glutamine-dependent asparagine synthase (ASN1, also called DIN6), and a mitochondrial substrate carrier family protein (MSCP). Their transcript abundance was determined by qRT-PCR with *n* = 3, presented as log 2 ± SE. Differences were not seen in the response to extended darkness.

*A. thaliana* line with T-DNA-insertion in the *ap2-2*-gene was identified in mutant collections. Selection of homozygous KO plants was confirmed at the DNA level by PCR using two genomic primers and one primer located on the T-DNA insertion. Two distinct bands were amplified in wild type and KO plant material indicating the absence of the wild type allele in the KO lines (Figure S.2B). Moreover the absence of *AP2-2*-transcript in the insertion lines was confirmed via sqRT-PCR (Figure S.2C). Under normal growth conditions (see Material & Methods) phenotypes of wild type and *ap2-2* were indistinguishable (not shown). The search for transcripts co-regulated with *AP2-2* in transcriptome databases allowed the identification of genes involved in metabolism, for instance AKIN beta1, the β-subunit of a 5′-AMP-activated protein kinase (At5g21170) involved in regulation of *N*- and *C*-metabolism [31], DIN10, a member of the raffinose synthase family (At5g20250), BXL1 (At5g49360) coding for a β-xylosidase, a glutamine-dependent asparagine synthase (ASN1, also called DIN6, At3g47340), and a mitochondrial substrate carrier family protein (MSCP, At1g72820) (Table S.1) (Figure 6). Fujiki *et al.* [32] observed up-regulation of ASN1 transcript in response to prolonged darkness. *AP2-2*-transcript increased in extended dark period of 5 h. This increased abundance decreased with longer dark phase extensions.

**Figure 7:**
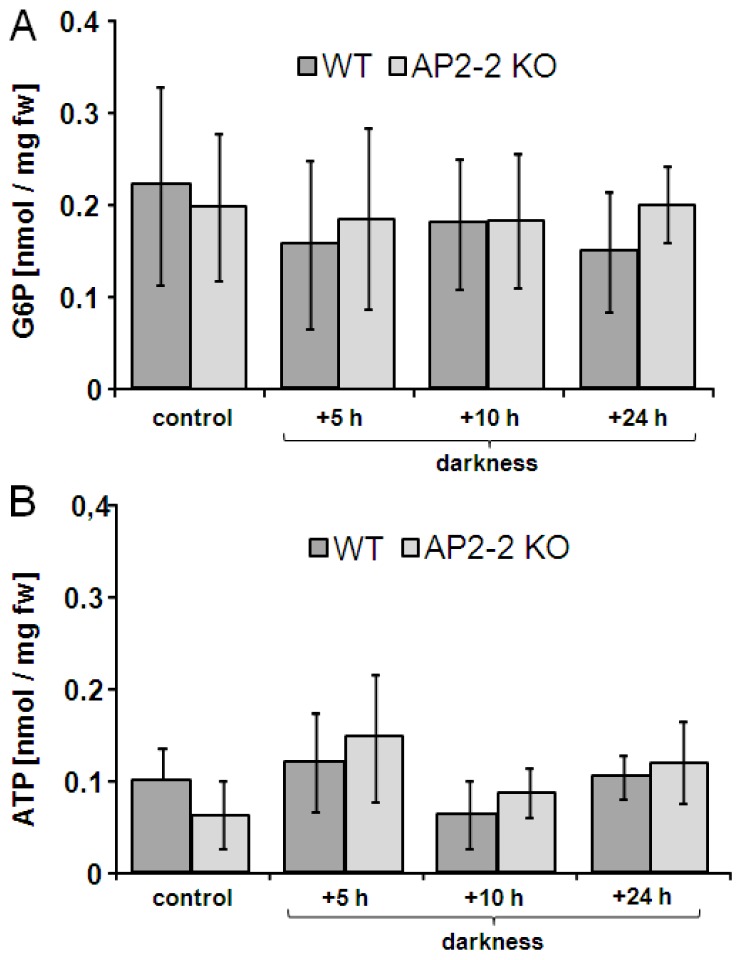
Glucose-6-phosphate (G6P) and ATP levels in WT and *ap2-2* KO after extended dark phase in wild type and *ap2-2* knock out plants. In parallel to transcript determinations (Figure 6) levels of ATP and G6P were quantified at 5, 10 and 24 h of extended darkness. Data are means of *n* = 3 experiments with 3 measurements each, ±SD.

The *AP2-2*-transcript was absent from the *ap2-2*-plants. Each of the co-regulated transcripts was up-regulated in plants exposed to extended darkness proving co-regulation. However, the transcriptional regulation was indistinguishable between *ap2-2* and wild type plants. The observation that *AP2-2*-transcript decreased under conditions of high assimilate contents and tentatively increased under conditions of low assimilate availability prompted us to perform a metabolome analysis (Figure S.3) and to determine ATP and G6P (Figure 7). A sampling time point after a dark period extended for 5 h was considered as appropriate experimental condition to compare *ap2-2* and wild type plants. However, leaf metabolite levels were not significantly different between mutant and wild type.

**Figure 8:**
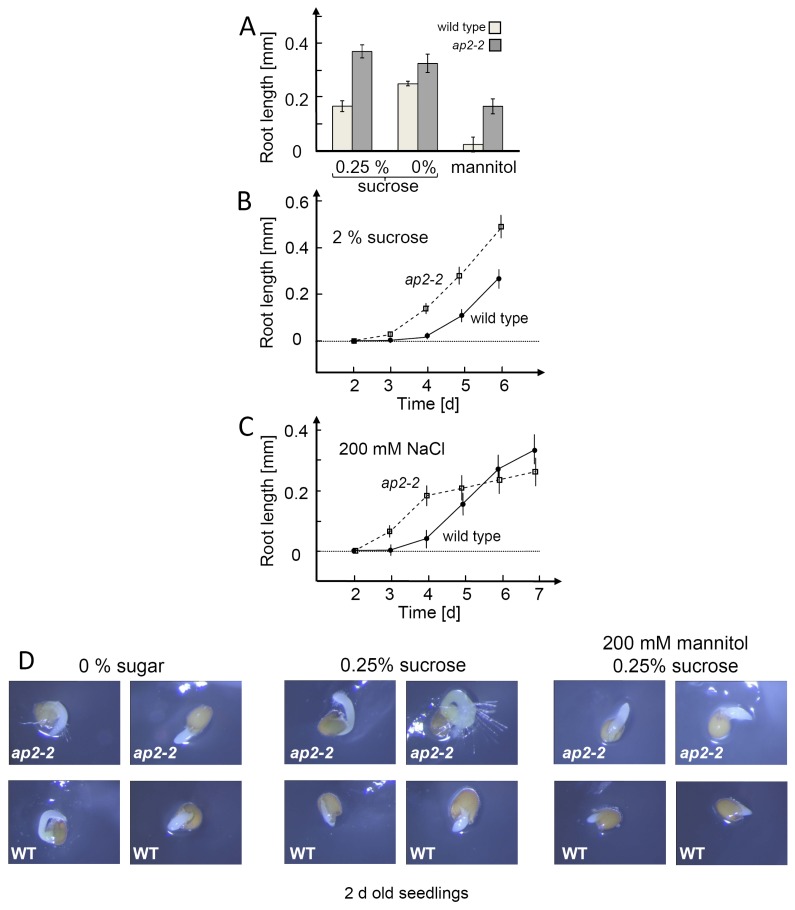
Root elongation of *ap2-2* knock out plants versus wild type under different treatments. (**A**) Root length of 2 days old *ap2-2* knock out or wild type seedlings under various treatments. Seeds were germinated in 0.25% sucrose/MS medium as control. Media were supplemented with 200 mM mannitol to induce osmotic stress or 200 mM NaCl in 0.25% sucrose to establish salt stress. Germination without sugar supplementation was also tested. The data were obtained from 60 seedlings and are presented as means ± SE; (**B**) Root development in 2% sucrose-containing MS medium. The data were obtained from 40 seedlings and are presented as means ± SE; (**C**) Root development in 200 mM NaCl. The data were obtained from 60 seedlings and are presented as means ± SE; (**D**) Comparison of 2 days old seedlings of wild type and *ap2-2* KO grown without sugars, 0.25% sucrose or 200 mM mannitol plus 0.25% sucrose.

The mutants were tested for changed germination and development under stress (Figure 8). Germination efficiency of *ap2-2* plants exceeded that of wild type in particular under osmotic stress induced by 200 mM mannitol. Seedling development was stimulated in the presence of 2% sucrose and initially also under salt stress. It should be noted that these differences occurred under conditions which almost completely suppressed or delayed seed germination and seedling growth, since under regular growth conditions the radicles of wild type and mutants reached 0.4 mm at day 2, while this value was only measured after 6 to 7 days under the stress treatments with high sugar (2%) or in 200 mM NaCl. Interestingly, salt stressed *ap2-2* slowed down growing at day 4, while wild type seedling continued to develop. Thus, at day 7, wild type seedlings were larger than *ap2-2*-plantlets.

## 3. Discussion

### 3.1. AP2/ERF-TFs Appear to Be Involved in Retrograde Signaling at Various Levels of Regulation

AP2/ERF transcription factors have been shown to control development [3,33,34] and modulate diverse stress responses [11]. Fifty-three AP2/ERFs were selected for an analysis in context of retrograde signaling based on their expression in leaves [28]. Transcript accumulation of most of the leaf-expressed 53 AP2/ERF-TFs was modified in response to more than one photosynthesis- or stress-related effector. Retrograde signaling [22–24] has been established as a multifactorial mechanism in the operation control of nuclear gene expression. Thus, there is no single readout for retrograde control. Instead, the pattern of transcriptional and posttranscriptional control defines the response. This work shows that many AP2/ERF-TFs are likely involved in retrograde adjustment of nuclear gene expression.

Cooperative or antagonistic regulation of AP2/ERF-TFs has been observed before. For example, CEJ1 is cooperatively regulated by ethylene and jasmonic acid [35]. ABI4 coordinates sugar-, ABA-, jasmonate- and ascorbate-linked redox signaling [36]. Likewise, DREB1A or DREB2 are considered as master transcription factors in abiotic stress responses including cold and drought [17]. The differential transcript regulation in response to six photosynthesis- and stress-related cues in leaves supports the conclusion that AP2/ERF-TFs are implicated in retrograde signaling pathways. Although the result does not resolve the functional hierarchy, it may be assumed that AP2/ERF-TFs function at three levels of regulation, (i) as master regulators such as RRTF1 [9]; (ii) as downstream signal executers and (iii) as combinatorial elements for response modulation. The latter two functions will be discussed further.

### 3.2. Unpredictable Behavior of Transcript Regulation from Single Effectors to Combinatorial Conditions

Single stressors have been applied to plant tissue in diverse experimental setups. However, there is a need to understand plant acclimation mechanisms including expressional regulation of AP2/ERF-TFs in response to combinations of simultaneously acting effectors. The plant hormone ABA modulates development, regulates seed germination and induces stress acclimation responses with global readjustment of gene expression. ABA-dependent signaling pathways have been deciphered in quite some detail [37]. In leaves, ABA is synthesized from violaxanthin via neoxanthin and xanthoxin in plastids. Xanthoxin is exported from the plastids and serves as precursor for ABA synthesis [38]. 9-*cis*-epoxycarotenoid dioxygenase (NCED) is a key enzyme in this conversion. Thus, ABA synthesis competes for the common substrate violaxanthin with the pathway of zeaxanthin synthesis for photochemical quenching. This reciprocal relationship and in addition the link of ABA to ROS stress may explain the *AP2/ERF* mRNA-transcript regulation observed upon feeding ABA to leaf discs. ABA accumulation indicates an imbalance in photochemical reactions and water status. In this line, ABA has been implicated in retrograde signaling in the H-light response from bundle sheath to the mesophyll gene expression system and coincides with a ROS signal [39]. Here, ABA altered transcript accumulation of many of the 53 leaf-expressed *AP2/ERFs* and each of the six selected AP2/ERF-TFs. The order of the stimulatory effect on transcript accumulation was *AP2-2* > *CEJ1* > *RAP2.5* > *ERF8* > *ERF11*, and a negative effect was detected for *ARF14*. Like ABA as an indicator of an imbalance in photochemistry, DCMU inhibits electron transfer from photosystem II to plastoquinone pool. As a consequence, plastoquinone, NADP and thioredoxin systems are oxidized. DCMU only stimulated AP2-2-expression, but with increasing strength suppressed transcript accumulation of CEJ1, ARF14, ERF8, RAP2.5 and ERF11 suggesting that there exists a link to either a reduction signal or singlet oxygen. DCMU enhances singlet oxygen generation at photosystem II [40]. However, this is likely to occur at H-light and may explain the antagonistic effect of H-light on ABA- and DCMU-induced *AP2-2* accumulation. H-light as reducing signal had no effect in three cases and in three other cases slightly inhibitory effect. Interestingly, H-light antagonized in each case the ABA stimulation. This may be explained by the usually reciprocal effect of efficient photochemical quenching with decreasing violaxanthin pool and ABA synthesis as described above. Apparently, the AP2/ERF-TFs respond to multiple signals and only combinatorial effects adequately describe the specific pattern realized in the leaves in a time- and condition-specific manner.

The six selected AP2/ERF-TFs belong to four different subclasses [6], namely ERF8, ERF11 and RAP2.5 to the ERF subgroup 1B, CEJ1 to the group DREB-A5, ARF14 to the RAV group and AP2-2 to the AP2-group. RAV-related ARF14 was identified as transcription factor containing the RLFGV-motif typical for active transcriptional repressors which are involved in phytohormone signaling and organ formation [41]. ERF8 interacts with ERF5 and is suggested to play a role in innate immunity and to take part in a signaling network controlling salicylic acid signaling [42]. CEJ1 identified in a screen for genes responding to both jasmonic acid and ethylene also responds to ABA as shown here [35]. The ABA-induced up-regulation of *ERF8* and *RAP2.5* was antagonized by DCMU, and that of *CEJ1* by H-light. Apparently, oxidative cues from plastoquinone in the presence of DCMU or reductive signals in the presence of H-light differentially feed into the pathways controlling transcript accumulation of these AP2/ERF-TFs. Interestingly, simultaneous application of all three effectors, namely ABA, DCMU and H-light which likely corresponds to a signal pattern achieved under extreme stress situations abolished the difference in mRNA level relative to control condition in three cases (*RAP2.5*, *ERF8*, *CEJ1*). The DCMU effect as suppressor dominated the combinatorial response of *ERF11* and *ARF14* indicating the requirement for a reduced electron transport chain as signal for maintenance of a normal transcript level of these TFs. The most peculiar transcriptional response was seen for *AP2-2* mRNA levels which behaved in a manner unpredictable from single cue administration. While H-light was able to suppress the ABA and DCMU effect, the triple cue condition revealed more than 20-fold up-regulation of *AP2-2*. It should be noted that in our hands the growth and experimental conditions needed to be tightly controlled for reliable reproduction of the data. These results support two important conclusions: (i) Behavior of transcriptional regulation as analyzed here for AP2/ERF-TFs cannot be extrapolated or predicted from regulatory features observed upon application of single stressors; (ii) AP2/ERF-TFs are part of regulatory networks [6,8]. The contribution of individual elements of the AP2/ERF-TF family to the whole system performance may not be unique or central to the function of the signaling network but can be compensated by others.

### 3.3. AP2-2 as an Element in a Shunt Circuitry in Starvation *Versus* Satiety Acclimation

AP2-2 was selected for the reason of its peculiar, strong and synergistic up-regulation in response to ABA and DCMU, and the antagonistic effect of H-light. The expected link to the metabolic status of the leaf tissue could be confirmed at the transcript level both in seedlings and in rosettes. 2% sucrose in the phytagel medium decreased the *AP2-2* mRNA level of seedling more than 3.5-fold in L-light and 2.7-fold in extended darkness. Transcript regulation of genes co-regulated with *AP2-2*, namely *AKIN beta1*, *ASN1*, *BXL1*, *DIN10* and *MSCP*, was undisturbed in *ap2-2*-knock out plants. They rather showed the wild type-like co-regulation with *AP2-2* in extended darkness. This indicates that transcript regulation of this group of genes is under control of an upstream signaling pathway and thus a common activation factor. The unaltered response of the co-regulated transcripts with known function in starvation even in the absence of *AP2-2* suggests that AP2-2 functions in a shunt pathway parallel to the co-regulated genes and is involved in downstream regulation of other unknown targets. Contento *et al*. [43] investigated transcriptome changes during sucrose starvation in suspension cultures of *A. thaliana* and observed expressional up-regulation of *AP2-2* after 24 and 48 h [43] supporting the assumed role for AP2-2 in acclimation processes during conditions of limitation in energy, reductant or more general in assimilates. Although a definite conclusion on the function is lacking, the germination assays tentatively suggest that AP2-2 may act as a suppressor, since the *ap2-2*-mutant germinated with higher efficiency under osmotic stress, salinity and high sugar. Surprisingly, the difference in germination was abolished in the absence of sugar. This phenotype may reflect the link between ABA and sugar also seen in the transcript regulation in the leaves. ABA and sugars are well-established regulators of germination as indicated by many genetic studies that identified elements involved in sugar and ABA dependent signaling, e.g., ABA insensitive *abi*-mutants and glucose-insensitive *gin*-mutants [44,45]. It may be concluded that AP2-2 functions in a general signaling context integrating stress input with assimilate availability both in seeds and leaves. The lack of a change in leaf phenotype, photosynthetic performance and metabolite profiles may be the consequence of redundancy. However, it is not unexpected that leaf-expressed AP2/ERF-TFs play a role not only in photosynthetic tissue under conditions that modify photosynthesis and involve retrograde control of nuclear gene expression but also in acclimation to stress of other organs.

## 4. Experimental Section

### 4.1. Plant growth and Treatments

*A. thaliana* Col-0 was grown in soil culture (1:1:1 mixture of Frühsdorfer Erde Klocke P, perlite and vermiculite) for 4.5 weeks under the following conditions: 10 h of light, 100 μmol quanta·m^−2^·s^−1^, 23 °C and 14 h darkness at 18 °C with 55% relative humidity. For low light acclimation 3 week old normal light grown plants were transferred to low light (8 μmol quanta·m^−2^·s^−1^) during the light phase and grown for further 10 days, and shown to be fully shade-acclimated [46]. For effector treatments, leaf discs (7 mm diameter) were floated upside down on 0.1 mM CaCl_2_. The medium was supplemented with ABA (1, 5, 10, 20 or 30 μM), 3-(3,4-dichlorophenyl)-1,1-dimethylurea (DCMU) (1, 5, 10 μM), 1 μM methylviologen (MV) or 30 mM sucrose, respectively, or the discs were illuminated with high light (800 μmol quanta m^−2^ s^−1^), or left in low light. After the 4 h treatment leaf discs were immediately frozen in liquid nitrogen. Heat stress was administered to intact leaves of 4.5 week old plants in a 30 °C water bath for 4 h. For the experiments with elongated dark phase, the plants were kept in complete darkness for 19 h after regular end of the previous light phase or in continuous light. Germination was measured on phytagel solidified Murashige Skoog medium supplemented with sucrose, NaCl or mannitol as indicated in the legend. Seed germination was measured every day using a standard microscope with imaging facility. Calibration was achieved using a microscope slide with calibrated object micrometer scale.

### 4.2. RNA-Isolation and cDNA Synthesis

RNA was isolated according to the method of Chomczynski and Sacchi [47] using Trizol reagents and 100 mg of frozen and pulverized plant tissue. After two Trizol and one chloroform extraction nucleic acids were precipitated with isopropanol overnight. Following two 70% ethanol washing steps the RNA pellet was dissolved in H_2_O. The subsequent cDNA-synthesis was performed as described in [48].

### 4.3. Transcript Profiling

The transcript levels of the TFs were quantified with sqRT-PCR as described previously [49]. Quantitative real time-PCR (qRT-PCR) analysis was done as outlined in [46]. See Table S.1 for sqRT- and qRT-PCR primer list.

### 4.4. Mutant Screening

To test for homozygous insertion, total DNA of *A. thaliana* wild type (Col-O) and a SALK line of AP2-2 (At1g79700) (SALK_018113) [50] was extracted from leaf material of 3 week old plants with 500 μL extraction buffer according to [51]. The isopropanol precipitates were dried in air and dissolved in 100 μL H_2_O. PCR was done with 5 μL of isolated DNA and a primer mixture of SALK left border T-DNA primer (LBb1.3) and appropriate gene-specific primers (94 °C 3 min; 35 × (94 °C 30 s, 58 °C 30 s, 72 °C 45 s); 72 °C 5 min) (see Table S.1 for primer list).

### 4.5. PSII Measurements

The effective quantum yield of PSII (ΦPSII) was measured during steady state photosynthesis using the Mini-PAM (WALZ, Effeltrich, Germany). Whole plants or leaf discs (7 mm diameter) were analyzed as described before [46].

### 4.6. Metabolite Analysis

Metabolite levels of *A. thaliana* wild type plants and *ap2-2* knock out plants were compared after extended darkness. Plant material was harvested and immediately frozen in liquid nitrogen. After grinding and following lyophilisation for 48 h, 7.7 mg of the plant material was used for the profiling by GC/MS according to [52]. Contents of glucose-6-phosphate (G6P) and ATP were determined spectrophotometrically using an enzyme assay with NADPH, glucose-6-phosphate dehydrogenase and hexokinase and recording of absorption at 340 nm. 150 mg of frozen plant material were extracted with 1 ml HClO_4_. 150 μL of K_2_CO_3_-neutralized supernatant were used for the enzymatic test [53].

## Supplementary Information



## Abbreviations

ABA: abscisic acid
*A. thaliana*: *Arabidopsis thaliana*
AP2: Apetala 2 transcription factor
Chl a: chlorophyll a
DCMU: 3-(3,4-dichlorophenyl)-1,1-dimethylurea
DREB: dehydration-response element binding protein
ERF: ethylene response factor
G6P: glucose-6-phosphate
H: high light (800 μmol quanta/m^2^s)
KO: knock out
L: low light (8 μmol quanta/m^2^s)
log-FC: logarithmic fold change
MV: methylviologen
N: normal light (80 μmol quanta/m^2^s)
RAP2: related to apetala 2
TF: transcription factor
