# Supplementary material for: Combinatorial Signal Integration by APETALA2/Ethylene Response Factor (ERF)-Transcription Factors and the Involvement of AP2-2 in Starvation Response
Source: Int J Mol Sci. 2012 May 16;13(5):5933–51. doi: 10.3390/ijms13055933 (PMC3382747; doi:10.3390/ijms13055933)
Supplement: Supplementary file 1 [file ijms-13-05933-s001.pdf]

Supplementary Information

Figure S.1. Effective photosynthetic quantum yield of leaf discs. ΦPSII in H-light decreased to about half within 1 h. No significant effect was observed in low light (**A**). Sucrose had no effect, while ABA slightly decreased ΦPSII (**B**). Application of 10 µM DCMU strongly decreased ΦPSII within 1 h. Methylviologen led to a slow but progressive decrease in ΦPSII (**C**). *n* = 3 with 6 leaf discs each, ±SE.

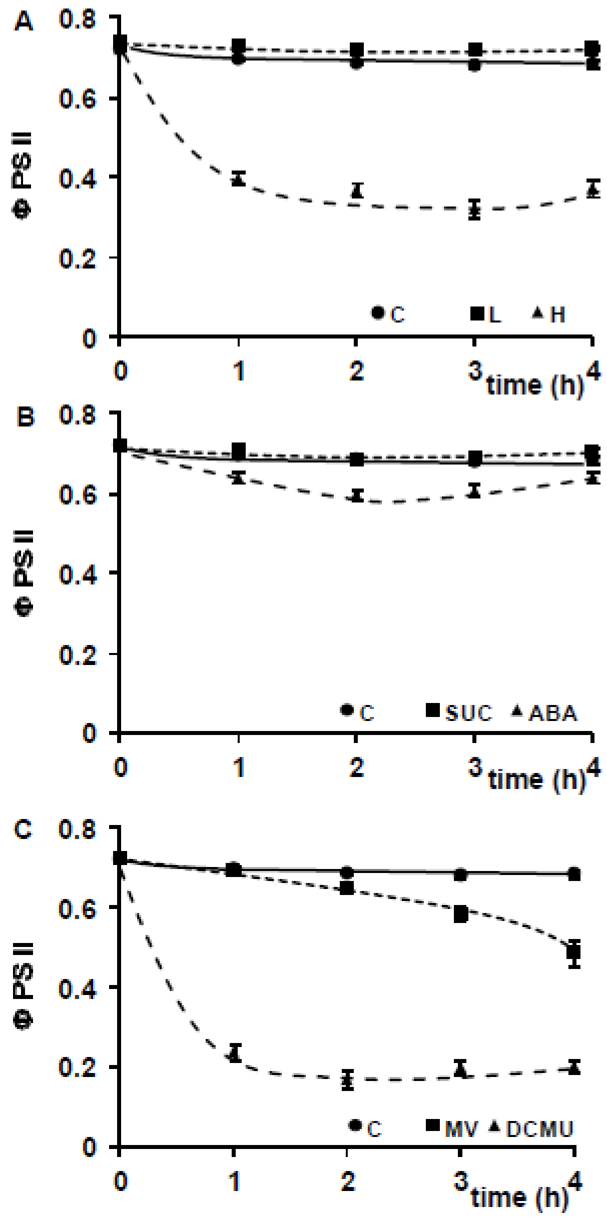

Figure S.2. Characterization of the *ap2-2* (At1g79700) KO-mutant. (**A**). The T-DNA insertion is located in the second exon of *ap2-2*. (**B**) The presence of the T-DNA insertion and homozygocity were confirmed by PCR with combinations of gene-specific and T-DNA insertion-specific primers (Table S.1) that generated a 1 kbp band in wild type or a 700 bp in *ap2-2* background. (**C**) Transcript analysis by RT-PCR gave the expected 220 bp product in the wild type and no products in *ap2-2*.

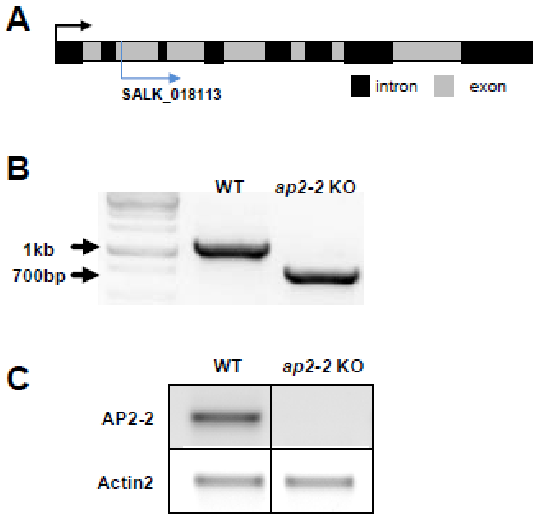

Figure S.3. Metabolite analysis of *ap2-2* and wild type measured in an extended darkness of 5 h. Metabolite profiles determined by GC-MS analysis were in three experiments. Significant differences were not observed.

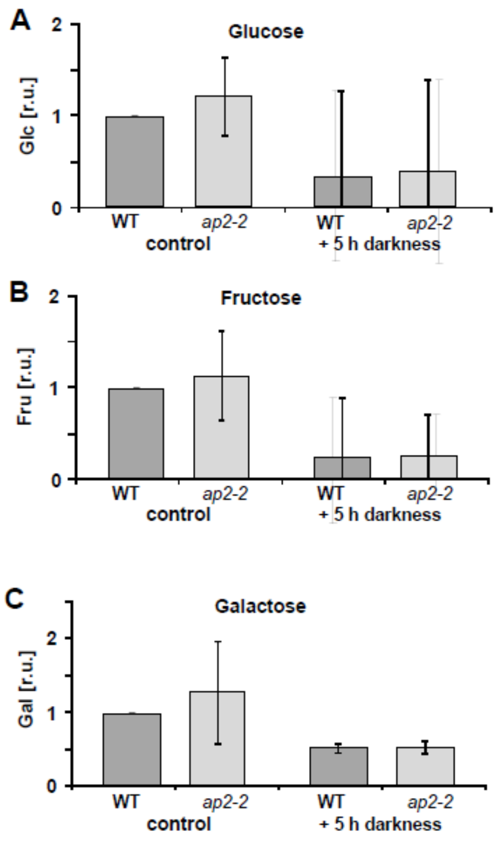

Table S.1. Primers for RT-PCR and SALK-T-DNA line of AP2-2.

Target Gene	ID Number	Forward Sequence (5’ – 3’)	Reverse Sequence (5’ – 3’)	T_A (℃)
ERF023	At1g01250	CAGCTGAATTTCCCTGAAGA	ACCCACCTCCACTCATCATA	52
RAV1	At1g13260	GATTCAGAGAACGGCGTAGA	CTTCGTCCATCTTCACGTCT	61
ADAP	At1g16060	TGGGTTTTCACGAGGTGTAT	CGGCTGATGTCGAAGTTAGT	55
ERF017	At1g19210	GACGTAACTTGTCGCGATCT	AACGATCACCGGAGTATTCA	58
ERF012	At1g21910	AACCATTTTGCCCCTACTTC	GCATCGTCGTCGAGTTTAGT	61
RAP2.13	At1g22190	TTCCCTGATCTCCGTCATAA	ATCTCCTCCTCCGTATCACC	58
ERF019	At1g22810	CGACGTTGCTTTCTTCTGTT	CCGCCCAGATAATCATACAC	61
TEM1	At1g25560	CCGATGAGTTTGAGCAGAGT	TAATCAAAACGCCTTTCGTC	63
ERF11	At1g28370	GCCTACGACAAACGTGCTAT	CACTCCTTTCATCGAACACC	58
RAP2.6	At1g43160	ATGGTGTCTATGCTGACTAATGTTG	AGACTGAAGTTGTATTGGGACAGAA	61
ERF3	At1g50640	CAGATTCAGAGGCGTAAGGA	GGGAACTGTTGTTGATGGTC	58
ERF8	At1g53170	GACCAATTTCGGTGTTATCG	CAGATCTAACGGCGGAGATA	58
RAP2.12	At1g53910	ATTTCGACGTCGGTGATGTT	TTCTAGCACCTTCCCTTGGA	61
CRF10	At1g68550	TAGGAAGAAGCCTGTTGGTG	CAGGTGAAGAACGAGAGCAT	61
RAP2.8	At1g68840	CGGCGATTTAGCTTTTCTTG	CACGAGACGGTTTAGCTTCC	58
HRE1	At1g72360	GAGCAGTCGGTACAAAGGAA	TCAGCCTCATGATTCTCCTC	61
MSCP	At1g72820	AGCTCTTTATCCTGCGGTTT	CAGCAGCTTTAGCCTCTGTC	56
DREB2D	At1g75490	CGAGATGTCATCATGTGGAA	AGTGGGGAATGAAAGGAATC	58
ERF013	At1g77640	GATCACCATCCATCTGCTTC	CATCGTTGCCTCTGAGCTAT	61
RAP2.4	At1g78080	GCGTTTTCTCTCCCTCCTCT	CTTTGGGCTGAGAAGATTCG	56
AP2-2	At1g79700	GTACTGGGGACGAGACACAC	CCCTTCCAATTCTAGCTTCC	61
DEAR3	At2g23340	GGAGGAAGAGGTGGTGATTT	CATCATCACCATCCGAATTT	58
ERF112	At2g33710	CTTCCCATGATCGCATTATC	TGTTCTTCACGTTTTCACCA	58
AP2-like	At2g41710	TGGAGAAGAATAACGGCAAG	TCTCTTGTGGGAGGTAGCTG	62
ERF034	At2g44940	ACCAACGGTGGAGATAAACA	CGGCTTGAATGTCTTTAGGA	52
CRF5	At2g46310	GGAAAGCGAAGAAAAAGTCC	CAACTGGGAATAACCAAACG	52
RAP2.5	At3g15210	ACCCACAATAATGCCAAGGA	GGACCTTCTGGTCACTCAGC	56
RAP2.3	At3g16770	GTGAAGAAGGAGCAGGCAAC	TATCACCACGGATCTGCTTG	61
Actin2	At3g18780	TTGGTAGGCCAAGACATCAT	GGAGCCTCGGTAAGAAGAAC	58
ARF14	At3g25730	TTAAAACGGCGGAGTTACTG	CTCCAACCTTTGGTCAACAC	61
CRF11	At3g25890	CAGATTCCTGAAATGGGTTG	GTGGTTCCAATGAGACCAAG	58
ASN1	At3g47340	TTGCTCGACACACGAGATAA	CTCATAAGGCGTTGAAGGAA	56
PKRP	At3g49160	TTAAAACCTGGTCCGTGTGT	ACAAACCCGGTACTGTTGAA	58
CEJ1	At3g50260	ACAAGCGTTCAAGACTTTGG	CCCCTATCGCATCTACTTGA	63
WRI1	At3g54320	ATCAGAATCTCGCGAATGAG	ACCGAGGTTGTTGTTGTTGT	61
SMZ	At3g54990	AAAATGCACCCAATTCAAAA	TGGACCGATTGATACCCCTA	52
ERF035	At3g60490	ATTATCCGGTTTGCTTCCTC	TCTTCGTTGCTTTCCTTGTC	61
AP2-like	At4g13040	TTCCAAATCCTAATGCCAGA	TCGCTTTCTTCTATGCTGCT	61
B-Amylase	At4g15210	TTCTGGGATTCACTGGTTGT	GTTCTCACCCGCAACTTCTA	56
ERF6	At4g17490	TGAAACCAAACCGGAAATAA	TCTCCTCTGCTGCTACAACC	58
ERF1A	At4g17500	GGAGCTAGGGTTTGGTTAGG	ACCGTCAATCCCTTATCCAT	58
CRF2	At4g23750	CACTCACCAAACCAGGATTC	AACTTCTTCTCTCCCGTCGT	61
ERF054	At4g28140	TCGTTAGAGGCTTTCCCTTT	CTTCGTTGCGATGTAAGGTT	61
ERF015	At4g31060	GCCTATGATGCTGCTTTGTT	CAGAGGAAGCAGGAACTTCA	58
RRTF1	At4g34410	GAAGGATGTCTCGGCTGTAA	ACACGTGTGGCTCTTTTAGG	61
FLO2	At4g36920	CAGACCCAATAACCCACTTG	ACTCTTTTTCAACGGCTGTG	56
ERF060	At4g39780	CATGACCGCTCAGAAACTCT	CCGTAGTATCCATCCTCGTG	61
ERF106	At5g07580	TGACCCCATCTCTAAAACCA	CCAAATCAGAGCTTTCCTCA	61
AIL6	At5g10510	GGAGGAAGAGTAGCGGTTTC	AAGACGTTTAGCTGCACCAC	58
TINY2	At5g11590	TATCGCGGAGTAAGGATGAG	CTGAATGTCTCGAGGGCTTA	63
RAP2.6L	At5g13330	AAAGGCACCAAGGCTAAACT	AAGAAGGCGTTGAAAAAGGT	58
LEP	At5g13910	TACAAGGTTTCTTGGGGTGA	GATCATTGCTAGGAGGAGCA	61
DIN10	At5g20250	GGAGAGTTTATGCAGCCTGA	TTAGCAAGCTGACACCATCA	61
AKINβ1	At5g22170	TACCAGCCGCAGAAGATTAC	GAGCGGTTTGTAGAGGACAA	58
ERF003	At5g25190	CGCTAAACTCCACAAATGCT	CGCTAAACTCCACAAATGCT	63
ERF5	At5g47230	CCGATGAAGGTGAGAAGAAA	CAACTGGGAATAACCAAACG	61
BXL1	At5g49360	CAAGATACGCGAGGACACTT	CGGGTCACTAGGTCTTGTTG	58
RD29a	At5g52310	GTGGAGTAGAGGAGCAACGA	CCGAACCATCCTTTAATCCT	58
ERF107	At5g61590	TGAGGAAAGCTCTGATTTGG	ACCTCAGGTGACGTTGTTGT	61
ERF104	At5g61600	TACAGGGGAGTGAGACGAAG	ACCCTTATCTCGCTTTCGTT	61
TOE3	At5g67180	TTAACGACTCACCCGATCAT	TGATATCTCCGGCTCTTCTG	58
ERF010	At5g67190	CACGGCGGTTTTCTATCTTA	CCGTTTTGATAATTCCGATG	61
SALK TDNA	Insertion	ATTTTGCCGATTTCGGAAC	-	58
SALK TDNA AP2-2	SALK_018113	CGTGACAGTGGACTAGACACG	AATTGCATTGGATTTGGACC	58

Table S.2. Co-expression data for AP2-2. The data were obtained from *Expression Angler* [S-1] and *Arabidopsis Co-expression data mining tool* [S-2].

ID	r-Value Expression Angler	r-Value Arabidopsis Co-expression Tool	Annotation
At3g15450	0,773	0,78543	Aluminium induced protein
At5g21170	0,754	0,795346	AKINBETA1
At5g22920	0,748	0,760928	CHY-type Zinc finger protein
At5g20250	0,692	0,693651	DIN10
At3g15630	0,687	0,68728	unknown protein
At4g35770	0,682	0,682269	ATSEN1
At2g17880	0,674	0,643799	Chaperone DnaJ-domain
At3g47340	0,672	0,685696	ASN1
At1g56220	0,652	0,658175	Dormancy/auxin associated family protein
At5g19120	0,649	0,699898	Eukaryotic aspartyl protease family protein
At1g80920	0,648	0,651041	Chaperone DnaJ-domain superfamily protein
At1g72820	0,646	0,728021	Mitochondrial substrate carrier family protein
At5g49360	0,645	0,682814	ATBXL1
At5g28770	0,644	0,666374	AtbZIP63
At1g03090	0,639	0,656148	MCCA
At2g25900	0,62	0,63037	ATCTH
At1g13700	0,615	0,606129	PGL1__6-phosphogluconolactonase 1
At3g02540	0,613	0,646452	Rad23 UV excision repair protein family
At3g26510	0,612	0,610532	Octicosapeptide family protein
At2g31810	0,612	0,620013	small subunit of acetolactate synthase protein
At2g20670	0,611	0,630448	unknown protein
At3g23050	0,599	0,588006	indole-3-acetic acid 7
At1g01620	0,596	0,606231	plasma membrane intrinsic protein 1C
At1g21830	0,596	0,653546	unknown protein
At5g24490	0,595	0,576504	30S ribosomal protein, putative
At3g60530	0,593	0,577982	GATA transcription factor 4
At1g25230	0,592	0,597163	Calcineurin-like metallo-phosphoesterase superfamily protein
At1g22740	0,592	0,603202	ATRABG3B
At1g68190	0,591	0,598229	B-box zinc finger family protein
At2g05540	0,588	0,607264	Glycine-rich protein family
At3g15450	0,773	0,78543	Aluminium induced protein

References

S-1. Toufighi, K.; Brady, S.M.; Austin, R.; Provart, N.J. The botany array resource: E-Northerns, expression angling and promotor analyses. *Plant J.* **2005**, *43*, 153–163.

S-2. Manfield, I.W.; Jen, C.H.; Pinney, J.W.; Michalopoulos, I.; Bradford, J.R.; Gilmartin, P.M.; Westhead, D.R. Arabidopsis Co-expression tool (ACT): Web server tools for microarray based gene expression analysis. *Nucleic Acids Res.* **2006**, *34*, W504–W509.

© 2012 by the authors; licensee MDPI, Basel, Switzerland. This article is an open access article distributed under the terms and conditions of the Creative Commons Attribution license (http://creativecommons.org/licenses/by/3.0/).
